# The Intrinsic Dynamics and Unfolding Process of an Antibody Fab Fragment Revealed by Elastic Network Model

**DOI:** 10.3390/ijms161226197

**Published:** 2015-12-11

**Authors:** Ji-Guo Su, Xiao Zhang, Xiao-Ming Han, Shu-Xin Zhao, Chun-Hua Li

**Affiliations:** 1 College of Science, Yanshan University, Qinhuangdao 066004, China; xiaozhangysuniv@163.com (X.Z.); a413140155@163.com (X.-M.H.); zsx360@yeah.net (S.-X.Z.); 2 College of Life Science and Bioengineering, Beijing University of Technology, Beijing 100024, China

**Keywords:** fab fragment of antibody McPC603, intrinsic dynamics, unfolding process, Gaussian network model, native structural topology

## Abstract

Antibodies have been increasingly used as pharmaceuticals in clinical treatment. Thermal stability and unfolding process are important properties that must be considered in antibody design. In this paper, the structure-encoded dynamical properties and the unfolding process of the Fab fragment of the phosphocholine-binding antibody McPC603 are investigated by use of the normal mode analysis of Gaussian network model (GNM). Firstly, the temperature factors for the residues of the protein were calculated with GNM and then compared with the experimental measurements. A good result was obtained, which provides the validity for the use of GNM to study the dynamical properties of the protein. Then, with this approach, the mean-square fluctuation (MSF) of the residues, as well as the MSF in the internal distance (MSFID) between all pairwise residues, was calculated to investigate the mobility and flexibility of the protein, respectively. It is found that the mobility and flexibility of the constant regions are higher than those of the variable regions, and the six complementarity-determining regions (CDRs) in the variable regions also exhibit relative large mobility and flexibility. The large amplitude motions of the CDRs are considered to be associated with the immune function of the antibody. In addition, the unfolding process of the protein was simulated by iterative use of the GNM. In our method, only the topology of protein native structure is taken into account, and the protein unfolding process is simulated through breaking the native contacts one by one according to the MSFID values between the residues. It is found that the flexible regions tend to unfold earlier. The sequence of the unfolding events obtained by our method is consistent with the hydrogen-deuterium exchange experimental results. Our studies imply that the unfolding behavior of the Fab fragment of antibody McPc603 is largely determined by the intrinsic dynamics of the protein.

## 1. Introduction

Due to their low toxicity and high efficiency, antibodies are growingly used as pharmaceuticals for the prevention and treatment of various diseases including cancer, infectious diseases, chronic inflammatory disease, and so on [1,2,3,4]. Thermal stability is one of the important issues that must be considered in antibody design. Partial unfolding may occur for the antibody with low thermal stability. The partial unfolded antibodies tend to aggregate, which results in a decrease in the antibody activity. Unfolding and aggregation are one of the major challenges that limit the antibody manufacture [5,6]. The investigation of antibody unfolding process is helpful for our understanding of the physical mechanism of antibody stability and provides valuable information for antibody design. Many experimental and theoretical studies have suggested that the native structure topology conceals a wealth of information about the folding/unfolding process of proteins [7,8,9]. In the present work, the structure-encoded dynamics and unfolding process of an antibody Fab fragment were studied by using a coarse-grained topology-based model, *i.e.*, elastic network model (ENM).

**Figure 1:**
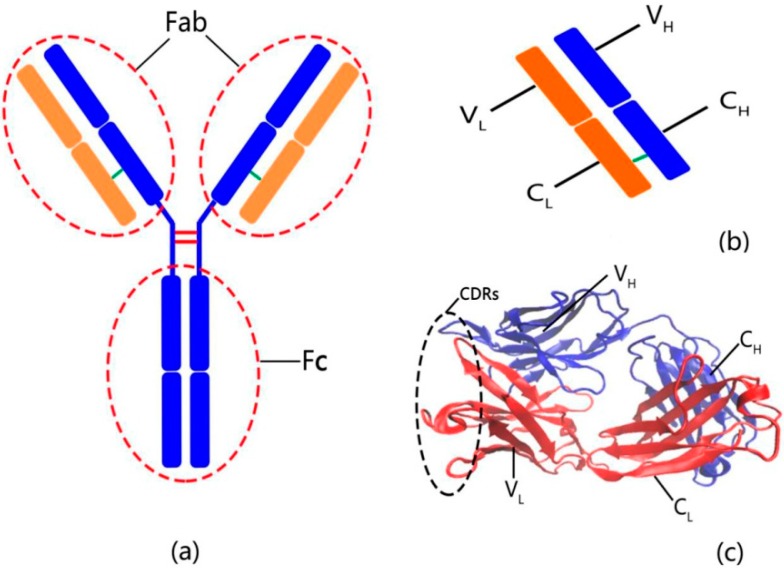
(**a**) The structure of immunoglobulin; (**b**) the simplified structure of Fab fragment; (**c**) the structure of McPC603 antibody.

The basic functional unit of an antibody is the immunoglobulin (Ig) monomer, which is a large Y-shaped protein produced by B-cells. The structure of Ig monomer is symmetrical, which consists of four polypeptide chains, *i.e.*, two heavy chains and two light chains linked together by disulfide bonds [10,11,12,13]. Each heavy chain contains a variable (*V*_H_) domain and several constant (*C*_H_) domains, and each light chain has one variable (*V*_L_) domain and one constant (*C*_L_) domain. According to the function of the antibody, the Ig molecular can be divided into three functionally distinct fragments: two identical antigen-binding fragments (Fabs) and one crystallizable fragment (Fc), as shown in Figure 1a. The Fc fragment is formed by paired C_H_ domains, which is responsible for the interactions with effector molecules and cells. The Fab fragments correspond to the two arms of the Y-shaped structure of the antibody, which are composed of the *V*_L_ and *C*_L_ domains of the light chains paired respectively with the *V*_H_ domain and one *C*_H_ domain of the heavy chains, as shown in Figure 1b. The two Fab fragments contain two identical antigen-binding sites that bind with antigens specifically. Each antigen-binding site is made up of three complementarity-determining regions (CDRs) in *V*_H_ and three CDRs in *V*_L_ (see Figure 1c), which is responsible for the antigens-binding specificity of the antibody. The structure of the Fab fragment of the phosphocholine-binding antibody McPC603 has been determined by X-ray crystallographic experiments [14].

Gaussian network model (GNM), which is one kind of ENM, is a simple yet effective method to investigate the dynamical properties encoded in protein structure [15,16]. GNM describes a protein tertiary structure as an elastic network, in which the sophisticated interactions between residues are represented by harmonic springs. Several studies have shown that such a simplified model is sufficient to describe the collective motions of proteins compared to molecular dynamics simulation and normal mode analysis with complex semiempirical force field, which indicates that protein intrinsic dynamics is largely determined by its structural topology [17,18,19]. GNM has a wide range of applications including investigation of protein functional motions [20,21], identification of functionally key residues [22], decomposition of subdomains of proteins [23], refinement of protein low-resolution crystal structures [24], and so on. It should be noted that GNM also has some limitations. The basic assumption in GNM is that the native structure is at the energy minimum, and only the native contacts are taken into account in this model. Therefore, GNM is not suitable to study partly misfolded states where non-native interactions may play important roles.

In this work, the structure-encoded dynamics and unfolding process of this protein were investigated by using GNM. Firstly, the temperature factors for the residues of the protein were calculated with GNM and then compared with the experimental measurements. Then, the mean-square fluctuation (MSF) of the residues in the protein, as well as the MSF in the internal distance (MSFID) between all pairwise residues, was calculated to evaluate the mobility and flexibility for different parts of the protein. Finally, the unfolding process of this protein was also investigated by using a GNM-based iterative method proposed by our group [25]. In our method, the unfolding process is mimicked through breaking the native contacts between residues one by one according to the fluctuations of the distance between them. The contact to be broken is chosen by minimizing the free-energy difference for the unfolding event. The protein unfolding process obtained by our method agrees well with the experimental observations and the molecular dynamics simulation results. Similar to our idea, several theoretical methods were proposed by other groups and successfully used to study the folding/unfolding process of proteins [26,27,28]. In the present work, the sequence of unfolding events for the Fab fragment of the antibody McPC603 was simulated with our proposed method and then compared with the available experimental observations.

## 2. Results and Discussion

### 2.1. The Comparison between the Computed and Experimental B-Factors

**Figure 2:**
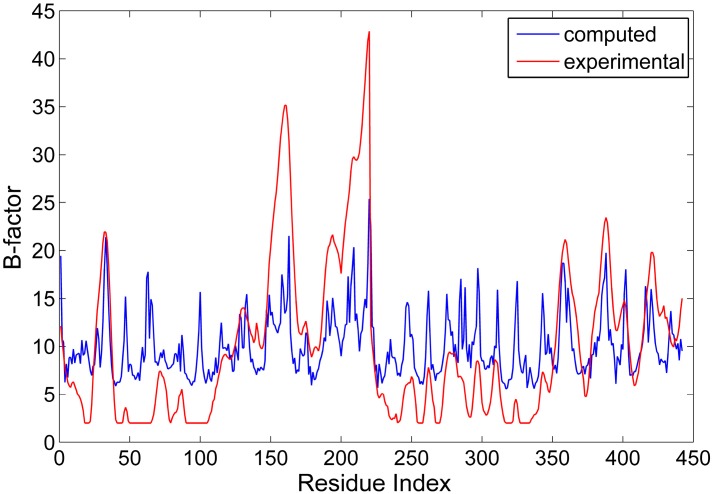
The comparison between the computed (blue line) and experimental (red line) B-factors for the Fab fragment of the antibody McPC603.

In the GNM, the spring coefficient γ is an adjustable parameter, which can be determined by normalizing the theoretical B-factor against the experimental one. The theoretical B-factor for all the residues in the protein was computed according to Equation (5), and compared with the data from X-ray crystallographic experiments. Then, the value of the spring coefficient was determined to be $0.751\;k_{\text{B}}T/{Å}^{2}$. Figure 2 displays the comparison between the computed and experimental B-factors. From this figure, it is found that the position of the peaks and valleys for these two curves agrees well with each other. The correlation coefficient between the computed and experimental data is 0.60, which is similar to the studies for other proteins [29]. Our results indicate that the GNM is applicable for investigating the intrinsic dynamics of the antibody McPC603 Fab fragment. It should be pointed out that our studies only concern with the relative fluctuations of residues, and the absolute value of the spring coefficient γ does not affect the results in this work. Therefore, our studies are also applicable to the proteins where the B-factors are not available in experiment.

### 2.2. The Intrinsic Collective Dynamics of the Fab Fragment of McPC603

**Figure 3:**
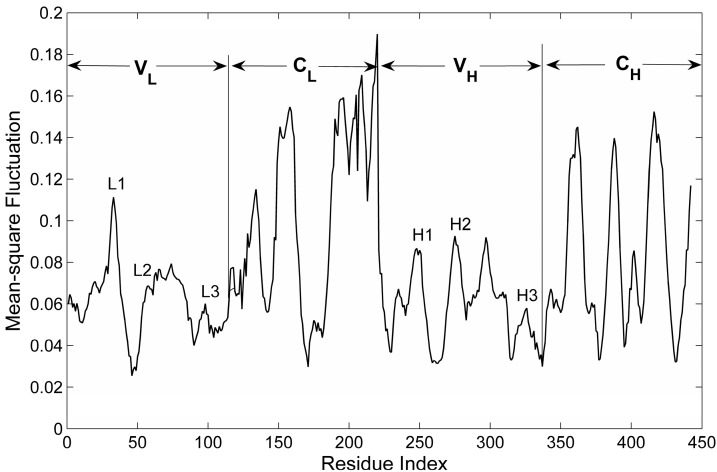
The root mean-square fluctuation of the residues for antibody McPC603 Fab fragment calculated by the first six slowest motions. The locations of the constant regions, variable regions and the six CDRs (*i.e.*, L1, L2, L3, H1, H2 and H3) are marked in the figure.

For proteins, the slow motion modes represent large-scale collective motions encoded in the protein structure [30]. In the present work, the first six slowest normal modes were considered in our calculation to reveal the collective dynamics of the Fab fragment of the antibody McPC603. The mean-square fluctuations (MSF) of the residues for the first six slow modes were calculated and shown in Figure 3. It is interesting to find that the constant regions both in the heave and light chains, *i.e.*, *C*_H_ and *C*_L_, exhibit distinctly larger MSF values than the variable regions, *i.e.*, *V*_H_ and *V*_L_, which indicates that the constant regions are more mobile than the variable regions. The X-ray crystallographic experiments have shown that the value of B-factors of the constant regions is obviously larger than that of the variable regions (see the red line in Figure 2). Our calculation result is consistent with the experimental data.

In the variable regions *V*_H_ and *V*_L_, it is found that the three CDRs in *V*_L_ (denoted as L1 (26–37), L2 (56–61) and L3 (97–102)) and three CDRs in *V*_H_ (represented by H1 (28–32), H2 (50–58) and H3 (102–109)) exhibit relatively large MSF values. These six CDRs form the antigen binding site. The large amplitude motions of the CDRs may be responsible for the binding of antigens with high specificity.

### 2.3. The Flexibility of Different Parts in the Structure of the Fab Fragment of McPC603

**Figure 4:**
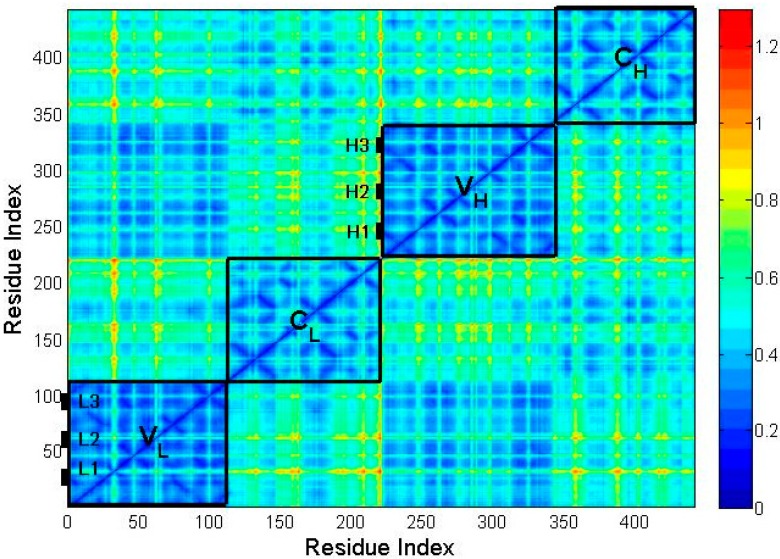
The mean-square fluctuation in the internal distance (MSFID) between all pairwise residues in different substructures of McPC603 Fab fragment, *i.e.*, *V*_L_, *V*_H_, *C*_L_ and *C*_H_. The three CDRs in *V*_L_ (denoted as L1, L2 and L3) and three CDRs in *V*_H_ (represented by H1, H2 and H3) are also marked in the figure. As shown in the color bar on the right, the blue regions in the figure indicate small MSFID values and the yellow-red regions represent large MSFID values.

The MSF value of residues only reflects the mobility of different parts in the studied protein. In order to investigate the flexibility for different substructures of the protein, the MSF in the internal distance (MSFID) between all pairwise residues in the substructures of the protein, *i.e.*, *V*_L_, *V*_H_, *C*_L_ and *C*_H_, were respectively calculated based on Equation (7). The smaller value of MSFID indicates more rigid motion of the corresponding residues. On the contrary, the higher MSFID value means more flexible residue motions. The calculation results are shown in Figure 4. In this figure, the blue color represents the lower value of MSFID and the yellow-red color corresponds to higher value. The four regions marked by rectangles along the diagonal of the figure correspond to the four domains of the protein, *i.e.*, *V*_L_, *C*_L_, *V*_H_ and *C*_H_. It is found that the MSFID values in the constant regions *C*_L_ and *C*_H_ are relatively larger than those in the variable regions *V*_L_ and *V*_H_, which implies that the constant regions are more flexible than the variable regions. The unfolding process of this protein discussed in the following section also showed that the constant regions unfold earlier than the variable regions due to their relative higher flexibility. Figure 4 also shows that in the variable regions, the six CDRs (L1, L2, L3, H1, H2 and H3) exhibit relative larger MSFID values, suggesting that these CDRs are relatively more flexible and unfold earlier than the other parts of the variable regions. Comparing Figure 3 with Figure 4, it is found that the substructures with higher mobility also have higher flexibility, which are disrupted at the earlier stage in the unfolding process.

### 3.4. The Unfolding Process for the Fab Fragment of McPC603

The X-ray crystallographic experiments showed that the structure of the Fab fragment of antibody McPC603 is almost symmetrical. The structures of *V*_L_ and *C*_L_ are respectively similar to those of *V*_H_ and *C*_H_, as shown in Figure 1. The folding/unfolding processes of *V*_H_ and *V*_L_ have been studied by hydrogen-deuterium exchange experiments. Both of these two domains contain an inner β-sheet and an outer β-sheet composed of several strands. In the pulsed exchange and folding competition experiments, the inner β-sheet of the *V*_L_ domain is formed early, but the *V*_H_ domain fails to form an extended nucleus in the early state of the folding reaction. It implies that the *V*_L_ domain folds before the *V*_H_ domain [31,32]. It is not clear why these two structural similar domains show differences in the stability and the folding/unfolding behavior. Freund and co-workers suggested that the reason may be due to the absence of some of the stable hydrogen bonds in the inner β-sheet of the *V*_H_ domain as compared to the *V*_L_ domain [31].

**Figure 5:**
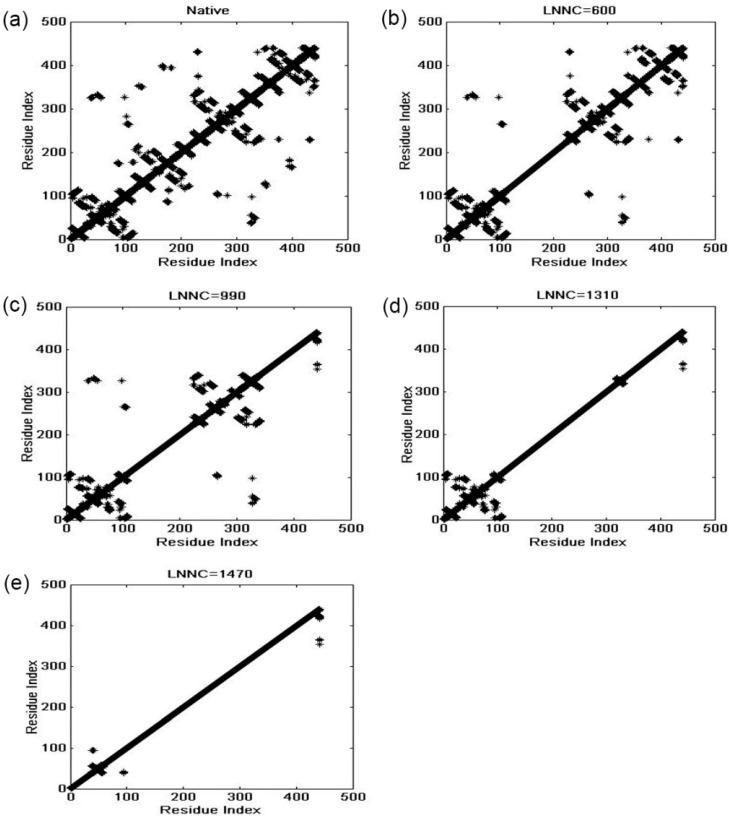
The sequence of unfolding process for the Fab fragment of McPC603. Pannels (**a**)–(**e**) display the contact maps of the native structure (**a**), and the snapshots with the loss number of native contact (LNNC) to be 600 (**b**), 990 (**c**), 1310 (**d**), and 1470 (**e**), respectively. In this figure, each native contact is represented by a black asterisk.

**Figure 6:**
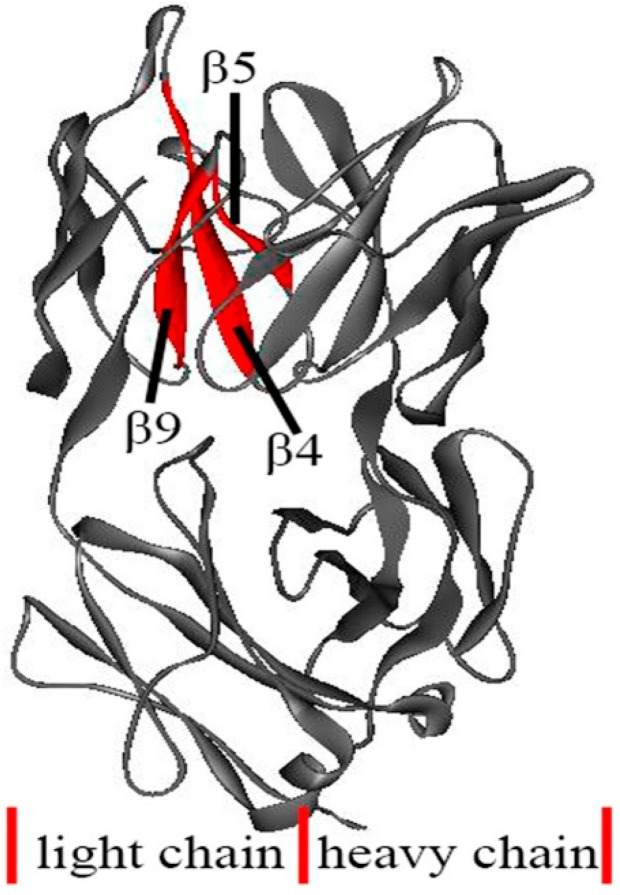
The most resistant substructures during protein unfolding process, which are β4, β5 and β9 in *V*_L_ domain marked by red color in the figure. The rest part of the protein is shown in gray ribbon model.

In the present work, the unfolding behavior of the antibody McPC603 Fab fragment is studied with the iterative method proposed by our group. The unfolding process of this protein was simulated by our method and the contact maps of several representative snapshots during unfolding process are shown in Figure 5. This figure presents the contact maps of the native structure (a), and the snapshots with the loss number of native contact (LNNC) to be 600 (b), 990 (c), 1310 (d), and 1470 (e), respectively. The unfolding process of the protein can be obtained from the change of the contact maps. The first unfolding event is the disappearance of the native contacts in *C*_L_ domain, as indicated by the change of the contact map from (a) to (b). Then, most of the native contacts in *C*_H_ domain are completely disrupted, as shown in panel (c). At this stage, the *V*_L_ and *V*_H_ domains still maintain their native structures. This result indicates that the constant regions *C*_L_ and *C*_H_ unfold earlier than the variable regions *V*_L_ and *V*_H_. This is consistent with the intrinsic dynamics of these domains discussed in the previous sections, in which the mobility and flexibility of the constant regions are larger than those of the variable regions. Roccatano *et al.* have also shown that the collective dynamics encoded in protein native structure largely determine the unfolding process of proteins [33]. From Figure 5c to 5d, most of the native contacts in *V*_H_ domain are lost, whereas the native contacts in *V*_L_ domain are maintained. The *V*_H_ domain is less stable and thus unfolds faster than *V*_L_ domain, which agree well with the hydrogen-deuterium exchange experiments. In our method, only the topology of native structure is taken into account. Therefore, the small topological difference between them may be the reason why these two structural similar domains show different stability during folding/unfolding processes of this protein. From Figure 5e, it is found that the most stable contacts in *V*_L_ domain are those formed by β-strand 4 (residues Leu39-Gln44), β-strand 5 (residues Lys51-Tyr55), and β-strand 9 (residues Ala90-Asn96) in the inner β-sheet, as displayed in Figure 6. The pulsed exchange and folding competition experiments also showed that these three β-strands folds quickly in the initial folding stage, which form the folding core of this protein. Our simulation results are consistent with the experimental observations.

**Figure 7:**
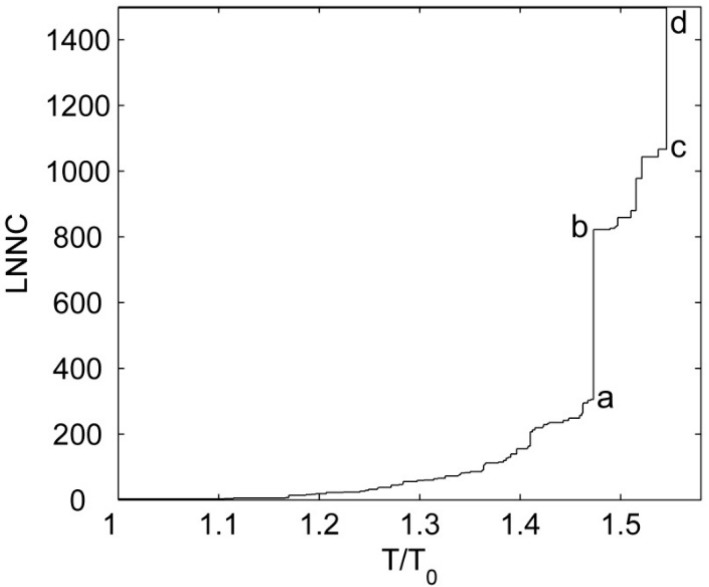
The value of LNNC as a function of the reduced temperature $T/T_{0}$. The different representative states are denoted by (**a**)–(**d**) in the figure.

Similar to the analysis of Srivastava and Granek [26], the cooperativity for the unfolding of McPC603 Fab fragment was also investigated in our study. Figure 7 displays the value of LNNC as a function of the reduced temperature $T/T_{0}$, where $T_{0}$ is the temperature corresponding to the rapture of the first native contact. It is found that there are several sudden jumps in the plot, at which several substructures in the protein are disrupted simultaneously. The jump from states (a) to (b), marked in Figure 7, corresponds to the cooperative unfolding of the constant regions *C*_L_ and *C*_H_, and the jump from states (c) to (d) represents the cooperative rapture of the variable regions *V*_L_ and *V*_H_. The experimental studies have also shown that both the constant regions and variable regions unfold in a cooperative, two-states manner [34,35]. Our simulation results are consistent with the experimental data. In our study, if the value of $T_{0}$ is assigned to be the room temperature, *i.e.*, 300 K, then the melt temperatures for the constant regions and variable regions can be estimated to be 442 and 464 K, respectively. The calculated melt temperatures are higher than the experimental results, which may be due to the neglect of the sophisticated interactions between residues in this coarse-grained model.

## 3. Materials and Methods

### 3.1. The Basic Principle of Gaussian Network Model

The Gaussian network model (GNM) is originally proposed by Flory and coworkers to study the fluctuation of elastic polymers [36]. Then, it was successfully used to reveal the intrinsic dynamics of proteins by Tirion at the atomic level [17], and by Bahar, Atilgan, Haliloglu and Erman at the amino-acid level [15,16,37]. In GNM, a protein is considered as an elastic network, in which each residue is reduced to a node at its Cα atom, and the residue pairs within a cutoff distance (here 7.3 Å is adopted) are connected by elastic springs. A uniform force constant is used for all these elastic springs. Based on this model, we can write the potential energy of the protein as

(1) $$V=\frac{1}{2}\text{γ}\left[\Delta R^{T}\left(\Gamma ⊗E\right)\Delta R\right]$$

where γ is the force constant of the springs in the model; {ΔR} is the 3N-dimensional column vector of the residue fluctuations; the superscript T represents the transpose of the column vector; E represents the three-order unit matrix; ⊗ is the direct product of matrices; Γ is the N-order symmetric matrix, called as Kirchhoff matrix, whose elements are expressed as

(2) $$\Gamma_{\text{ij}}=\{\begin{array}{cc} -1 & if\;\text{i}\neq \text{j}\;and\;R_{\text{ij}}\leq r_{\text{c}}\\ 0 & if\;\text{i}\neq \text{j}\;and\;R_{\text{ij}}>r_{\text{c}}\;\\ -\sum_{\text{i},\text{j}\neq \text{i}}\Gamma_{\text{ij}} & if\;\text{i}=\text{j} \end{array}$$

Here, $R_{\text{ij}}$ is the separation between the ${\text{i}}^{th}$ and ${\text{j}}^{th}$ residues in the protein and $r_{\text{c}}$ is the cutoff distance.

The cross-correlation between the fluctuations of two residues in the protein can be calculated by

(3) $$\langle \Delta R_{\text{i}}\Delta R_{\text{j}}\rangle =\frac{k_{\text{B}}T}{\text{γ}}{\left[\Gamma^{-1}\right]}_{\text{ij}}$$

where i and j respectively represent the ${\text{i}}^{th}$ and ${\text{j}}^{th}$ residues, $k_{\text{B}}$ is Bolthmann constant, and T is the absolute temperature. When i=j, the mean-square fluctuation of the ${\text{i}}^{th}$ residue can be obtained as

(4) $$\langle {\left(\Delta R_{\text{i}}\right)}^{2}\rangle =\frac{k_{\text{B}}T}{\text{γ}}{\left[\Gamma^{-1}\right]}_{\text{ii}}$$

According to Debye-Waller theory, the B-factor of the ${\text{i}}^{th}$ residue can be written as

(5) $$B_{\text{i}}=\frac{8\pi^{2}}{3}\langle {\left(\Delta R_{\text{i}}\right)}^{2}\rangle =\frac{8\pi^{2}k_{\text{B}}T}{3\text{γ}}{\left[\Gamma^{-1}\right]}_{\text{ii}}$$

The inverse of the Kirchhoff matrix can be calculated by eigen-decomposition method given by

(6) $$\Gamma^{-1}=U\Lambda^{-1}U^{\text{T}}$$

Here, U is a orthogonal matrix whose column $u_{\text{i}}\left(1\leq i\leq \text{N}\right)$ is the eigenvector of Γ, and Λ is a diagonal matrix whose diagonal element ${\text{λ}}_{\text{i}}$ is the eigenvalue of Γ. There are one eigenvector with zero eigenvalue, which corresponds to the overall translation of the protein. The rest N−1 eigenvectors represent the motion modes within the protein structure. The modes with low eigenvalues represent the collective motions, which are usually relevant to protein function. While the modes with large eigenvalues correspond to the geometric irregularity in the protein structure.

In this work, in order to investigate the flexibility for different substructures of the protein, the mean-square fluctuation in the internal distance (MSFID) between all pairwise residues of the protein was calculated. The MSFID between the ${\text{i}}^{th}$ and ${\text{j}}^{th}$ residues can be computed by [38,39]

(7) $$\begin{array}{l} \langle {\left(\Delta R_{\text{ij}}\right)}^{2}\rangle =\langle {\left(\Delta R_{\text{i}}-\Delta R_{\text{j}}\right)}^{2}\rangle =\langle {\left(\Delta R_{\text{i}}\right)}^{2}\rangle +\langle {\left(\Delta R_{\text{i}}\right)}^{2}\rangle -2\langle \Delta R_{\text{i}}\Delta R_{\text{j}}\rangle \\ =\frac{3k_{\text{B}}T}{\text{γ}}\left({\left[\Gamma^{-1}\right]}_{\text{ii}}+{\left[\Gamma^{-1}\right]}_{\text{jj}}-2{\left[\Gamma^{-1}\right]}_{\text{ij}}\right) \end{array}$$

### 3.2. The Iterative Unfolding Method

We have proposed a GNM-based iterative method to study protein unfolding process [25]. In our method, the unfolding process of proteins is simulated through breaking the native contacts one by one according to the fluctuation in the internal distance between the residues. The fluctuation of the internal distance between all pairwise residues was calculated according to Equation (7).

Our method mimics the way in which these native contacts break in response to slowly increasing temperature. The native contacts are expected to break in a fluctuation-dependent manner, and the nonlinear elasticity during protein unfolding is considered by iterative normal mode calculations. The protein unfolding process was mimicked by using the following procedure:

I. According to Equation (7), the values of MSFID for all residue pairs in the protein are calculated based on the topology of native structure.

II. The residue pair with the largest MSFID value is disrupted, and then a new structural topology, corresponding to a newΓ matrix, is obtained.

III. Based on the new Γ matrix, the MSFID values for all residue pairs are recalculated by using Equation (7).

IV. The above steps (III) and (IV) are iterated until all the non-bonded contacts in the protein are disrupted.

V. All the structural topologies obtained by the above method are collected to reproduce the unfolding process of the protein.

## 4. Conclusions

Antibodies play important roles in the immune system of living organisms. Currently, antibodies have been growingly used as pharmaceuticals in the clinical treatment of various diseases. In antibody design, the thermal stability as well as unfolding and aggregation are important issues that must be considered. The investigation of antibody unfolding process can provide valuable information for antibody design. Many studies have indicated that the native structural topology largely determines the folding/unfolding process of proteins. In the present work, the intrinsic dynamical properties and the unfolding process for the Fab fragment of antibody McPC603 were investigated by use of a topology-based model, *i.e.*, Gaussian network model (GNM).

In order to reveal the intrinsic dynamics encoded in protein structure, the mean-square fluctuation (MSF) of the residues in the protein was calculated by the sum of first six low-frequency modes. It is found that the constant regions exhibit larger MSF values than the variable regions, implying more mobile motions for the constant regions compared to the variable regions. In the variable regions, the six complementarity-determining regions (CDRs) also have relative large MSF values. The large amplitude motions of the CDRs are considered to be associated with the immune function of the antibody.

Then, the mean-square fluctuation in the internal distance (MSFID) between all pairwise residues was calculated to investigate the flexibility of different substructures in the protein. The calculation results show that the MSFID values in the constant regions are relatively larger than those in the variable regions, and the six CDRs in the variable regions also exhibit larger MSFID values. These flexible regions tend to unfold earlier during the unfolding process of the protein.

At last, the unfolding process of the antibody McPC603 Fab fragment was simulated by the iterative method proposed by our group. Our simulation results show that *C*_L_ domain unfolds first, which is followed by the unfolding of *C*_H_ domain. At this stage, the *V*_L_ and *V*_H_ domains still maintain their native structures. Then, the native structure of *V*_H_ domain is disrupted. The most resistant part is the substructure formed by β-strands 4, 5 and 9 in the inner β-sheet of *V*_L_ domain. Our results show that the flexible regions tend to unfold earlier during the unfolding process. The sequence of the unfolding events obtained by our method is consistent with the pulsed hydrogen-deuterium exchange and folding competition experiments results. Our studies indicate that the unfolding behavior of the antibody McPc603 Fab fragment is largely determined by the intrinsic dynamics encoded in the native structure of the protein.

The Fab fragment of antibody McPC603 is an all-β protein. Our method can successfully reproduce the unfolding process of this kind of protein structures. Besides that, our previous study has indicated that this method is also suited to the proteins with mixed α/β structures [25]. Certainly, the generality of our method should be further examined by more proteins with various kinds of structures.
